# Realising the potential of correlates of protection for vaccine development, licensure and use: short summary

**DOI:** 10.1038/s41541-024-00872-6

**Published:** 2024-04-29

**Authors:** Deborah F. King, Helen Groves, Charlie Weller, Ian Jones, Jakob P. Cramer, Peter B. Gilbert, David Goldblatt, Marion F. Gruber, Beate Kampmann, Diadié Maïga, Marcela F. Pasetti, Stanley A. Plotkin, Alexander Precioso, Liya Wassie, Frederick Wittke, David C. Kaslow

**Affiliations:** 1 Wellcome, 215 Euston Road, London, NW1 2BE UK; 2 WHO consultant, Jinja Publishing Ltd, London, UK; 3 Coalition for Epidemic Preparedness Innovations (CEPI), 215 Euston Road, London, NW1 2BE UK; 4 Vaccine Infectious Disease Division, Fred Hutchinson Cancer Center, Seattle, WA USA; 5 Great Ormond Street Institute of Child Health, University College London, London, UK; 6 International AIDS Vaccine Initiative (IAVI), 125 Broad Street, New York, NY 10004 USA; 7 London School of Hygiene & Tropical Medicine (LSHTM) Keppel Street, London, WC1E 7HT United Kingdom; 8 World Health Organization (WHO) - Regional Office for Africa, Cité du Djoué, P.O.Box 06, Brazzaville, Republic of Congo; 9 Center for Vaccine Development, University of Maryland School of Medicine, Baltimore, MD USA; 10 University of Pennsylvania, Philadelphia, PA USA; 11 Instituto Butantan1500, Vital Brasil Avenue, Butantã, São Paulo, SP 05503900 Brazil; 12 Armauer Hansen Research Institute, Jimma Road, ALERT Campus, Addis Ababa, Ethiopia; 13 MSD, The Circle 66, Zürich, CH Switzerland; 14 PATH 2201 Westlake Avenue, Suite 200, Seattle, WA 98121 USA; 15 Present Address: US FDA, Seattle, WA 98121 USA

**Keywords:** Vaccines, Adaptive immunity

## Abstract

On 27–29^th^ of September 2022, Wellcome convened an international multi-stakeholder workshop to discuss the use of Correlates of Protection (CoP) to accelerate vaccine development, the hybrid format meeting was attended by 80 delegates including developers, manufacturers, regulators, public health officials and policy-makers from 17 countries, including 7 LMIC’s.

Discussions focused on the perspectives of key stakeholder groups, including academic investigators, vaccine manufacturers, regulators and policymakers, to define the purpose and requirements of CoP data. Experts concluded that CoP can support decision-making throughout development, licensure and policy. Specific pathogen case studies were used to illustrate the current evidence and potential applications of CoP.

Lack of standardisation in sample collection, assay choice and data analysis were identified as key barriers for the discovery and use of CoP, especially in early stages of development. Collaboration between stakeholders to agree on the use of standardised protocols, reagents and assays and approaches to harmonise data analysis, would enable development of more reproducible and comparable data sets. Significant gaps were noted for non-serological data, particularly the role of mucosal and T-cell immunity which remain poorly understood, and where lack of standardisation in sampling and analyses along with lack of appropriate data analysis tools were highlighted as an issue.

It was noted that regulators do consider CoP data in licensing applications, as part of a wider data package, but typically assess their use on a case-by-case basis, and that the evidence needs of policy-makers may extend beyond data needed for licensure to inform implementation decisions. Participants identified the need for a rigorous framework to evaluate the strength of evidence of a biomarker as a correlate of protection, to support data prioritisation and decision-making.

Workshop discussion identified the following barriers to the use of CoP data in vaccine development:

1. Lack of consistency in data collection, analysis and presentation hampered the use of CoP data. There was an urgent need to standardise approaches to develop robust data sets at earlier stages of development, requiring co-ordination and collaboration between stakeholders.
2. Further guidance is needed on the type and strength of evidence required to enable the use of CoP data for decision-making throughout development including in licensure packages and post licensure in policy and implementation decisions.
3. Knowledge of what constitutes protection at relevant mucosal surfaces was lacking for most diseases and further research is needed to define the current status of mucosal immune responses, how they are measured, their relationship to systemic immune responses and how this data relates to vaccine effectiveness.
4. The contribution of cellular immune responses to protective immunity was poorly understood, and challenging to measure reproducibly due to their diverse and divergent nature at population level. Developing tools to enable clinical validation of cellular immune responses is needed.

Here we summarise the key challenges and opportunities in defining data requirements to establish CoP early and enable their use throughout vaccine development, licensure and implementation, with recommendations to address evidence gaps and advance the field of correlates discovery research.

## Introduction

Vaccines have had a significant impact on global morbidity and mortality over the last century, with estimates indicating that millions of lives are saved each year as a result of vaccination^1,2^. There are over 30 vaccine preventable diseases including those that contribute significantly to childhood mortality such as measles, but new vaccines are needed for diseases such as tuberculosis (TB), HIV, and emerging infectious diseases such as Sudan Ebolavirus, and Lassa virus. Vaccine development, particularly late-stage development is costly, time-consuming and is associated with high risk of candidate failure, with as few as 10% of vaccine candidates achieving licensure after launch of phase 2 trials^3,4^. An average vaccine candidate takes 10 years and costs $500 m to develop^5–7^. The high level of investment needed to progress vaccine candidates through late-stage development combined with high risk of failure can disincentivize development of products, especially where there is limited or unknown commercial market, which is the case for many diseases that affect LMIC’s^8^ or when products do not have a clear pathway to demonstrate efficacy.

Research published by Wellcome in 2021^9^ defined barriers to vaccine development, particularly focussing on late-stage development of vaccines for emerging infectious diseases, diseases disproportionately affecting low-income countries, and pathogens associated with antimicrobial resistance (AMR). A key finding from this report was that lack of correlates of protection was a systemic barrier to vaccine development. Improving our understanding of the nature and use of CoP has the potential to reduce the level of risk by improving go/no-go decision-making in vaccine development and thus reduce the time and cost of clinical development. This also has the potential to provide a pathway to licensure for products for which efficacy testing is unfeasible due to low or unpredictable incidence e.g. Zika and Chikungunya viruses or where very large trials would be needed to establish efficacy e.g. maternal vaccines for prevention of Group B streptococcus disease^10,11^.

To address this issue, Wellcome convened an international multi-stakeholder workshop to discuss the use of CoP to accelerate vaccine development and licensure by (1) defining the overarching data requirements needed to enable early discovery of CoP and their use throughout development, licensure and vaccine introduction and effectiveness monitoring, (2) identify barriers to discovery and use of CoP data and (3) identify opportunities for research that would advance the field.

### Essential background

A correlate of protection is broadly defined as an immune marker predictive of protection against a specified clinical disease endpoint. Multiple immune mechanisms can be responsible for clinical protection including serum and mucosal B-cell-mediated responses (e.g. neutralising and binding antibodies, functional antibody responses, B-cell subsets, across different Ig classes – IgA, IgG, IgM), T-cell-mediated responses including memory responses (across multiple T-cell subsets), cytokines and other mediators (including those associated with T-cell or B-cell responses), and innate immune responses. The relevance and contribution of these mechanisms varies by pathogen. For some pathogens, such as poliovirus, antibodies are the main mediators of protection; less commonly, for example for varicella-zoster virus and shingles, cell-mediated immunity is the key protective mechanism^12^. In many cases, multiple and potentially interdependent immune mechanisms likely contribute to protection. A further point to consider is that protective mechanisms, even if effective, can be overcome by high doses of infectious agent.

For each pathogen, mechanisms of protection may be different at different stages of infection and for different indications or endpoints. COVID-19 provides an example: Whilst there is no validated CoP for prevention from infection at mucosal surfaces, neutralising antibodies and T cells may provide protection in the upper airway, whereas protection in the lungs includes the role of binding antibodies through Fc, anamnestic antibodies and T cells, with the most severe outcomes of COVID-19 likely to require multiple branches of adaptive immunity acting synergistically^13,14^. Correlates of protection may also differ between populations, for example because of past exposure to infections, or by age (e.g. efficacy of rotavirus vaccines in LMIC settings^15^. Finally, for most pathogens, the concept of a protective threshold gives a misleading picture of the relationship between a biomarker and absence of illness. In reality, there is a continuous relationship between biomarker levels and degrees of clinical protection, rather than a ‘cliff-edge’. New statistical approaches could enable regulators to move away from a threshold model towards one that better reflects this relationship.

The level of knowledge about CoP varies by pathogen, and this has implications for decision-making. Sources of data from which CoP can be identified include: Studies of natural history of infection, analysis of immune responses during clinical trials, passive transfer studies (e.g. using monoclonal antibodies), investigation of vaccine failures (breakthrough infections), controlled human infection studies, extrapolation from animal models. In the absence of CoP, vaccine efficacy studies are required. However, as the level of evidence towards a CoP increases, such data can support decision-making, for example serum antibody thresholds are well-established as CoPs for vaccines against encapsulated bacteria e.g. Haemophilus influenzae type b, Meningitis C, Streptococcus pneumoniae^16–19^ and have been used as the basis for regulatory approvals as part of the data package. Factors that impact the utility of the data package include: the quality and relevance of samples analysed, the status of the assay used to generate the data, whether functional responses are assessed, and how data are presented e.g. with reference to international standards. The latter is not a regulatory requirement, but it is essential to make comparisons across studies to build confidence in CoPs across data sets. Development of a body of CoP data including thresholds for protection is an iterative and consultative process and may take many years.

### Definitions

For the workshop, the broad definition that a CoP is an immune marker predictive of protection against a specified clinical disease endpoint was used. Discussion focussed on their potential to act as predictors of vaccine efficacy, and therefore guide decision-making in clinical development, licensure and use. The strategies used to demonstrate vaccine effectiveness and the contexts in which CoP data may be used were recently described by Gruber et al.^20^ depending on how well established the relationship between the CoP and the clinical endpoint, the availability of licenced vaccines to act as comparators and the need for post-marketing studies to confirm clinical benefit. These strategies for demonstrating effectiveness were used to frame the discussion of the challenges for development of vaccines against influenza, COVID-19, TB, Group A streptococcus (GAS), Group B Streptococcus (GBS), Nipah, and filoviruses (Table 1), with consideration on how CoP could support development decisions when no licenced comparator vaccines exist to enable clinical immunobridging, and where efficacy studies were unfeasible.

**Table 1:** Use case for correlates of protection data in establishing vaccine effectiveness

Context of use	Licenced comparator vaccine needed?	Post-marketing study required to confirm benefit?	Diseases targeted (biomarker)
Randomised controlled trial using clinical disease endpoint.	No	No	Dengue, malaria, Tuberculosis, Shingles, Respiratory syncytial virus
Immunobridging using scientifically well-established biomarker (with threshold titre)	Yes	No	Hepatitis B (Anti-HBs >10 mIU/ml) Haemophilus influenzae type b (conjugate) (Anti-Hib 0.15 μg/ml) Pneumococcus (Anti capsular polysaccharide Ab 0.20–0.35 μg/ml)
Immunobridging using biomarker reasonably likely to predict protection (with threshold titre)	Yes	Yes	Influenza (HI antibody titre >1–40)
Immunobridging using biomarker for demonstrating effectiveness using animal studies	No, animal and clinical trials to bridge vx-induced immune response in animals to humans using biomarkers	Yes	Anthrax (anti-toxin Ab) Zaire Ebola (anti-glycoprotein binding Ab)
Immunobridging using biomarker without threshold	Yes	Maybe	SARS-CoV-2 (neutralising antibody)
Vaccine for which biomarker is needed to infer effectiveness in the absence of a licenced comparator vaccines and unfeasible efficacy studies.	No	Yes	Nipah, Zika, Chikungunya, Group A Streptococcus, Group B Streptococcus

### Defining stakeholders, uses and data requirements for CoP data

Whilst CoP data can be informative in vaccine development, sample collection and data analysis are inconsistent, and the way in which data are used and the contexts in which it is considered by regulators and decision makers is poorly understood. To improve consistency and collaboration between vaccine stakeholders, we drafted a framework which would collate the uses and requirements of CoP data for all stakeholders. The framework was modelled on the data purpose matrix developed by Moore et al. for prioritisation of burden of disease (BoD) data^21^. The BoD framework of Moore et al., was adapted to address three vaccine objectives: Vaccine development, regulatory requirements and licensure, and vaccine policy. For each objective the key stakeholders were identified, the way in which CoP data could be used were considered and finally we explored the underlying requirements of CoP data; which data were needed, how should it be collected, and the required properties of such data to make it usable in decision-making by stakeholders. The initial draft identified these needs at a pathogen agnostic level (Table 2) and this framework was applied to Group B Streptococcus as an illustrative example (Table 3).

**Table 2:** Data purpose matrix for correlates of protection data (disease agnostic)

	Vaccine objective		
	Clinical development	Regulatory and licensure	Vaccine policy and introduction
Key stakeholder/audience User of the CoP data	Clinicians Epidemiologists, microbiologists, immunologists Statisticians Manufacturers/Developers (pharma and biotech, PPPs) Funders and donors Consortium members	NRAs and Regional Regulatory Authorities WHO Vaccines Pre-Q Manufactures/Developers (pharma and biotech, PPPs) Funders and donors	WHO, SAGE, GNN RITAGs NITAGs Ministries of Health/Finance Industry/manufacturers
Data purpose: What do the stakeholders use the CoP data for? What decisions are made using the CoP data?	Identify correct/best choice of vaccine antigen based on pathogen biology Confirm lot-to-lot consistency Confirm lack of interference in concomitant use Provide early insight into efficacy Enable design of go/no-go criteria for Ph1 to determine progress to Ph2 De-risk or down-size phase 3, Extend indication through the use of immunobridging to expand label claims e.g. into additional age groups, Validate success of tech transfer Inform formulation, schedule and dose. To establish biomarkers for Immunobridging.	Immunobridging of a new product by comparing immune responses to a licenced comparator to infer effectiveness in: - Different age groups or demographic groups, - Change of dose, regimen or need for boosters - Change in formulation - Bridge manufacturing changes - Establish lack of interference in concomitant vaccine use Inclusion of additional strains to a licenced product without efficacy data Essential part of data package where efficacy studies are not ethical or feasible Definition of endpoints for phase IV evaluation if required.	To reduce delays in vaccine introduction by establishing immunogenicity in local populations where direct efficacy data are not available. Inform design of phase 4 studies to gather safety and effectiveness data in local populations and link to immunogenicity (and validate correlate). Prioritising limited vaccine stocks to key target groups Refining dosing or boosting regime Determine susceptibility of population to disease where a threshold is established. duction
Requirements for the use of CoP data Data requirements: What data, from where/when (disease- or endpoint specific) Properties of data: What do the stakeholders need to use CoP data with confidence as part of a data package	Consistent sampling collection and processing methods (especially for mucosal immunity) Availability of accurate, standardised, validated assays including: agreement on performance criteria (QC), endpoints, and where non-functional assays would be acceptable Specific T cell assays (cheaper and more easily standardised) using standardised reagents. Globally used & validated standards – ideally international standards - and reagents e.g. common source of antigen Framework for assessing quality of data and evidence Standardised and validated analysis methods inc. statistical plans “Breadth” studies to see suitability of CoP across serotypes and populations	Consistent use of assays, reagents, and standardised procedures to produce reliable and comparable immunogenicity data. Clarity and consensus on definition and use of CoP/ immune biomarkers between regulators, especially in immunobridging. Well-designed dose-ranging studies Framework for assessing quality of CoP data and evidence Coordination to enable inter-regulatory reliance through transparency Consistent documentation	Post-licensure studies to assess durability and effectiveness of CoP in different populations Data from multiple clinical trial sites to give confidence that CoP reflects immune responses of where vaccine will be used Alignment with regulators on what constitutes sufficient “protection” data Guidance from SAGE Communication and co-ordination with regulators and developers to clarify data needs for decision-making. Guidance needed on when it is appropriate to use CoP data for an intended purpose, and what evidence requirements are.

*NRA* National regulatory authority, *SAGE* Scientific Advisory Group of Experts, *GNN* Global NITAG network, *RITAG* Regional Immunisation Technical Advisory Group, *NITAG* National Immunisation Technical Advisory Group.

**Table 3:** Data purpose matrix for correlates of protection data for Group B Streptococcus vaccines

	Vaccine objective		
	Clinical development	Regulatory and licensure	Vaccine policy and introduction
Key stakeholder/audience User of the CoP data	Academic researchers Manufacturers/developers (pharma and biotech, PPPs) Funders and donors Consortium members	NRAs WHO vaccines pre-Q Manufacturers/developers (pharma and biotech, PPPs) Funders and donors	WHO, SAGE, GNN RITAGs NITAGs Ministries of Health/Finance Industry/manufacturers Health care workers Gavi
Data purpose: What do the stakeholders use the CoP data for? What decisions are made using the CoP data?	**Identify biomarker of interest and establish association with outcome of interest e.g early onset vs late onset disease** **Use CoP to advance vaccine development by informing Go/No-go decisions** **Identify promising candidates for support** Attract investment in promising vaccine candidates for late-stage development **Design clinical study to demonstrate effectiveness based upon CoP: protocol approval in advance of vaccine licensure.**	**Defining pathway to licensure in the absence of clinical efficacy for neonatal invasive GBS disease.** **Conditional approval of vaccine in the absence of demonstrated clinical efficacy/effectiveness against the desired clinical endpoint** **Definition of endpoints for phase IV evaluation if part of regulatory approval requirement.** **Approval of clinical study to demonstrate effectiveness based upon CoP; protocol approval in advance of vaccine licensure.** **CoP to persuade regulators that if achieved by vaccine would prevent early- and late-onset disease in infants (already framework agreed with FDA, concern remains about derivation of CoP)**	**Speeding up implementation or reducing ‘dead time’ between vaccine approval and policy recommendation:** **Inform study design of phase 4 effectiveness study including validation of CoP as predictive biomarker (may be supported by Gavi)** **Define strategy for licensing second-generation vaccines using CoP alone** **FVVA** – now published and a framework for new data to feed into for value proposition and policy decision-making Beyond direct protection: **Ancillary data about wider clinical benefits for health economic models** Aspirational but useful for stronger prioritisation? Info may not be on label? (data to feed into FVVA)
Data requirements: - *What CoP data (what biomarker for what outcome)* - *When and where (sample site) to collect CoP data* - *How to measure and validate the CoP data* *Format or form of communication that enables decision-making*	**For a defined biomarker e.g. anti-capsular IgG** Serum samples at delivery and up to 90 days post birth Rectovaginal swabs Cord and maternal blood at delivery GBS strain Quantitive measurement of Ab responses and association with GBS disease. Functional antibody responses **Evaluation of data for derivation of CoP using harmonised assay to allow for:** **- Universal CoP (geographically diverse populations studied)** **- Bridging for different valencies of vaccines** **Support developing international reference standards (lessons learnt from PCV)**	**Harmonised data collected from representative populations across multiple geographies, serotypes to create aggregate CoP** **TPP refinement: Single dose agreed but CoP could help understand need for booster and booster scheduling.**	**Phase IV post licensure studies to support vaccine introduction:** Vaccine probe studies to observe prevention of carriage or acquisition (including serotype replacement, adult disease in addition to preterm labour and stillbirth as an outcome of vaccine use). **Linkage of CoP with reduction of acquisition during pregnancy important:** Small risk of vaccine driving replacement disease. **Epidemiological studies across representative geographical locations required: burden data is still lacking for many geographies:** 90% infant death due first 24 h – data often missed. **Epi studies across representative geographical locations required: burden data still lacking for many geographies:** good epidemiological data exist for SA and some other African countries, India, UK, USA. **Epidemiological data gaps in Latin America, NZ, AUS.** Need to establish what epidemiological data are seen to be sufficient in regions/countries.

*NRA* National regulatory authority, *SAGE* Scientific Advisory Group of Experts, *GNN* Global NITAG network, *RITAG* Regional Immunisation Technical Advisory Group, *NITAG* National Immunisation Technical Advisory Group, *FVVA* Full Value Vaccine Assessment.

### Use of CoP in clinical development

Vaccine developers identified that CoP could be used to guide the choice of antigen and platform, and help to optimise vaccine formulation, dose, dosing schedule and choice of adjuvant at early stages of vaccine development. They can also be used to validate success of technology transfers and confirm lot-to-lot consistency by manufacturers.

Of particular value is the potential of CoP’s to de-risk phase III trials, by facilitating earlier demonstration of proof of concept. This was illustrated by the development of a respiratory syncytial virus (RSV) vaccine, which used CoP to guide development of a maternal vaccine to protect against severe illness in infants. Data on palivizumab, a monoclonal antibody known to protect infants against RSV, was used to derive an antibody threshold associated with protection^22^. Maternal responses in phase I studies were greatly in excess of this threshold, providing confidence to move to proof-of-concept studies^23^. Modelling was used to identify the fold-rise in neutralising antibody titres needed in pregnancy to provide protection in infants at different ages, and a subsequent proof-of-concept study in pregnant women demonstrated high levels of vaccine efficacy, consistent with modelling, and provided confidence to move forward to a phase III study^24^. Such a programme relies on being able to model the relationship between the biomarker and the endpoint, and the relative strength of the association.

However, the difficulty associated with validating the relationship between the proposed CoP and clinical benefit early in clinical development was exemplified by the case of the *Staphylococcus aureus* vaccine SA4Ag. The vaccine was designed to neutralise key virulence factors and elicited good neutralising antibody levels in animal models and phase I studies^25^. However, a phase IIb study failed to demonstrate efficacy, indicating that the putative CoP were not strongly associated with protection in humans. Better understanding of natural immunity, such as that derived from natural history studies may help to address such issues. Similarly, although an HIV mosaic vaccine was shown to be protective in animal challenge models and immunogenic in a phase IIa study, a phase III study failed to demonstrate efficacy, indicating the difficulty in developing animal challenge models that are predictive of vaccine efficacy in phase III field studies and deriving conclusions from animal models that would apply to humans^26^.

The contrasting outcomes between RSV and *S. aureus*, and HIV examples demonstrate one of the challenges of using CoP data: how to establish a CoP as a reliable predictor of efficacy early in clinical development, so that it may effectively guide clinical development decisions. A key consideration is how to evaluate the strength of evidence that a CoP can predict clinical benefit. The development of unbiased methods that evaluate the relative strength of evidence from different data sources and provide a tool to reliably establish the association between the biomarker and clinical benefit, could improve the accuracy of criteria for clinical development decisions and to assess the overall strength of the data package.

Developers concluded that CoPs can play a critical role in de-risking vaccine development, particularly by providing early evidence of proof of concept, and can be used at multiple other points in the clinical development pathway, however, a reliance on unvalidated CoPs can lead to phase IIB/phase III failures.

### Use of CoP in regulatory and licensure decisions

#### General regulatory considerations

**Fig. 1:**
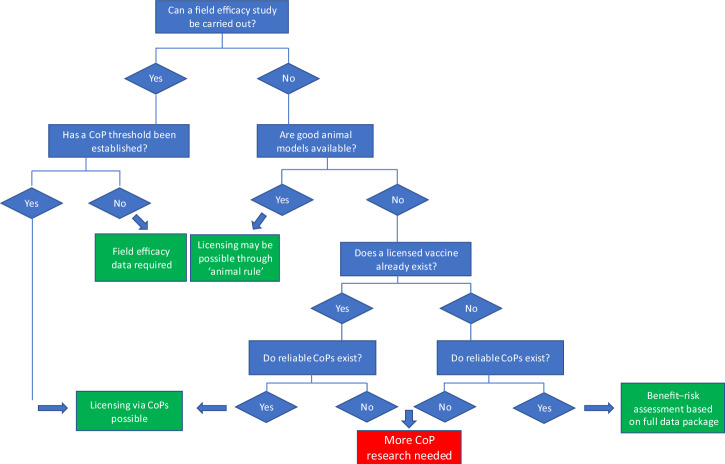
Simplified decision tree for consideration of correlates data in initial licensing of a vaccine.

The second vaccine objective defined was vaccine regulation and licensure, with stakeholders including national and regional regulatory authorities, developers, manufacturers and the WHO pre-qualification team. To approve a product, regulators must have confidence in its safety, quality and efficacy. Safety and quality standards are well established for vaccine development and were not the subject of discussion. The conventional way to assess efficacy is through phase III field studies, and if reasonably feasible, these will likely be required to support licensure applications. Feasibility of a phase III clinical trial combines both epidemiological and economic feasibility, and while the two are linked (in the simplest way, trials studying disease endpoints which are frequently observed are likely to be achievable relatively quickly, and with lower cost, than those with less frequently observed outcomes) there are some diseases for which the predicted time to achieve efficacy data based on a clinical endpoint are unfeasible e.g. a cluster-randomised ring vaccination trial for a Nipah virus vaccine was estimated to take 516 years and over 163,000 vaccine doses under current epidemic conditions^27^. Similarly, estimates to establish efficacy of a maternal Group B Streptococcus vaccine would require enrolment of up to 80,000 participants, making the trial unfeasibly large^10^. However, regulators stressed that they consider data packages including both clinical and pre-clinical data, and when considering data packages that include CoP data, they are weighing up whether immunogenicity data can be used to accurately and comprehensively predict efficacy in a proposed new use of a vaccine. The role that CoP data can play in initial licencing of a vaccine is summarised in a decision tree (Fig. 1).The greater confidence they have in the reliability of CoP to accurately infer vaccine effectiveness with acceptable residual uncertainty, the more likely they are to consider them in licensure applications. The more similar a proposed new use of a vaccine is to its existing use, the more confidence there is likely to be that the relationship between a CoP and efficacy is maintained. Interpretation of CoP data is not made by regulators alone; advisory committees are typically engaged to develop a consensus position. Regulators also consider how feasible it is to collect efficacy data and the benefit–risk perspective of the consequences of not supporting authorisation and providing access to a vaccine, particularly relevant for severe infections with limited treatment options and no existing vaccine, when a higher level of residual uncertainty around CoP’s may be tolerable. Residual uncertainty over the benefit of such products will likely require conduct of confirmatory post-marketing studies, in order to fully establish effectiveness. Because of these issues, regulators typically take a case-by-case approach that considers the nature of the pathogen and its epidemiology, current prevention and treatment options, and the level of supporting evidence that a CoP is a reliable predictor of efficacy. It is also important to note that policy and practice varies widely between regulatory authorities in relation to use of CoP data and licencing decisions.

#### Inference of effectiveness through Immunobridging

CoP data can be used to establish biomarkers to inform strategies to demonstrate vaccine effectiveness through clinical immunobridging: The inference of effectiveness based on comparison of immune responses, where immunogenicity endpoints from a new product, formulation or population are compared to those achieved in a study with known efficacy. In such strategies it is implied that the immune endpoint will predict other important components of the immune response^28^. Immunobridging is a well-established strategy to infer vaccine effectiveness using defined biomarkers under conditions not studied in a clinical efficacy endpoint trial such as different age or demographic groups, dose levels or dosing regimens, formulations of the same vaccine (e.g. when including additional antigens), to bridge changes in manufacturing processes and to evaluate immune interference when a product is co-administered with other vaccines. Immunobridging strategies rely on a suitable comparator vaccine with known efficacy data being available. Examples of immunobridging for COVID-19 vaccines include extension of COVID-19 vaccination to younger age groups, recommendations for boosting after primary series due to a decline in neutralising antibody levels and licensure of new COVID vaccines based on a different platform than the licensed comparator. There are also notable examples of immunobridging being used to approve a new vaccine for the first time, such as EMA’s recommendation of a monovalent vaccine against SARS-CoV-2 beta strain as a booster based on neutralising antibody titre as the primary endpoint^29^.

#### Inference of effectiveness through the use of established correlates for protection

The weight of evidence of a CoP will vary between pathogens, with some having established antibody thresholds associated with protection against infection or disease e.g. HepB vaccines, whilst others may have an established association between an immune response and protection but without threshold.

Protective antibody thresholds have been established for encapsulated bacteria, such as *Haemophilus influenzae* type b (Hib), *Neisseria meningitidis*, *Streptococcus pneumoniae* and used in vaccine licensure decisions. For Pneumococcal vaccines, anti-capsular antibodies were identified as central to protection against pneumococcal disease and a 7-valent pneumococcal conjugate vaccines (PCV) demonstrated high levels of efficacy in the USA^30^. Studies of serum antibody concentrations and vaccine efficacy were used to derive an initial antibody threshold for protection of 0.2 μg/ml^31^, and a consensus threshold figure of 0.35 μg/ml was eventually established reflecting results from additional clinical trials in different settings^17^. This threshold was incorporated into WHO guidance, which established that a new vaccine could be licensed using the serological CoP of 0.35 µg/ml and has been used in licensing of additional pneumococcal vaccines, including increasingly multivalent products.

Some regulators are willing to consider CoP data for certain decisions even if a threshold has not been established, as long as there is strong supportive evidence of the relationship between a correlate of protection and efficacy, so that it is reasonably likely that a biomarker is predictive of clinical benefit. For example, licencing of some COVID-19 vaccines such as bivalent vaccines targeting original and omicron variants was based on the clear relationship between neutralising antibody levels and protection against symptomatic disease, even though a protective threshold was not defined^32^.

Where a CoP is well-established, new vaccine products can pursue licensure via an accelerated approval route even when no licensed comparator products are available, through conducting an immunogenicity trial against a surrogate endpoint based on an immune marker, and conduct further post-marketing studies to establish effectiveness e.g. seasonal influenza vaccines^33^.

#### Establishment of correlates of protection where efficacy studies are unfeasible

The strategies for gaining regulatory approval of a vaccine outlined above rely on availability either an approved comparator vaccine or established biomarkers associated with protection. The focus of several case study pathogens discussed at the workshop focused on the scenario of how to progress development when efficacy trials are unfeasible, and there is no suitable comparator available e.g. when a vaccine is being developed for the first time against a specific disease endpoint. Phase III studies may be unfeasible because the disease endpoint is uncommon and would require very large numbers of participants (e.g. neonatal invasive GBS disease), take many years to develop (e.g. Rheumatic heart disease following episodes of rheumatic fever caused by GAS) or because cases occur during sporadic and unpredictable outbreaks (e.g. filovirus infections such as Ebolavirus disease). Approval of vaccines in the absence of classical efficacy data represents a challenge to developers, however, it is possible to infer effectiveness using immunological endpoints in certain circumstances e.g. when a product is intended for use against serious or life-threatening conditions.

To address the challenge of evaluating clinical benefit in absence of human efficacy data, Janssen’s Ebolavirus vaccine was licensed by bridging immune responses in vaccine recipients to survival after Ebola Virus challenge in a stringent animal model. This required selection of an immunological marker associated with protection in a lethal animal model. The relationship between EBOV neutralising antibodies, glycoprotein binding antibodies, and glycoprotein reactive T-cells with survival in a non-human primate model was assessed, and the binding antibody assay was identified as the having the strongest correlation with survival. An ELISA was then used to measure binding antibody responses in human vaccine recipients and compared to animal data. A survival probability curve for different levels of antibody response was generated and used to infer vaccine efficacy^34^. This was sufficient to support licensing. In this context, the FDA established the “Animal Rule”, which allows for licensure of biological products when human efficacy studies are not ethical and field trials to study the effectiveness are not feasible, a vaccine against anthrax was the first to be approved under these regulation in 2015^35^. Another recent example is the approval of live attenuated vaccine to prevent Chikungunya virus (CHIKV) disease by the US FDA. The phase 3 study measured neutralising CHIKV antibody titres above a threshold indicative of protection, which had been established in non-human primate passive transfer studies as a surrogate of protection reasonably likely to predict clinical benefit^36,37^.

When vaccine effectiveness studies are approved based on a biomarker-based endpoints, phase IV post-licensing effectiveness studies are likely to be a requirement in order to validate effectiveness, and are a potentially powerful approach for assessing vaccine effectiveness in real-life contexts and can generate data to validate CoP. Such a requirement for confirmatory clinical studies can be required by regulatory authorities when products are approved via an accelerated approval pathway. However, one drawback is that many low- and middle-income countries (LMICs) lack the infrastructure to conduct comprehensive phase IV studies.

#### CoP data requirements for regulators

In terms of data requirements, critical factors from the regulatory perspective were the reliability and comparability of the immunogenicity data presented. Functional data are generally preferred but of utmost importance is the consistent use of assays and standardisation of reagents and procedures, so that data can be pooled and meaningfully compared. When no comparator vaccine is available, standardisation of assays becomes particularly important, as data being compared are derived from different studies and laboratories (unlike most clinical immunobridging studies, where data on a test vaccine and a licensed comparator are obtained in the same study using the same assay). Use of international standards is not mandatory for regulatory decision-making but can harmonise and further aid the interpretation of data.

Because of the many nuances and need for a case-by-case approach, regulatory authorities recommend that developers engage at an early stage with regulators to discuss the role that CoP data could play in licensing decision-making, as well as associated evidence needs. The case-by-case approach can be a drawback from the perspective of developers, who would benefit from additional general guidance. This approach also has the potential to create inconsistencies in decision-making between regulatory authorities regarding the data package needed to support use of a biomarker to demonstrate vaccine effectiveness for a specific pathogen. It also does not indicate what steps developers could take to generate more compelling data. It can also lead to divergence in opinion across regulators, with some having a greater appetite for CoP data than others. This could be addressed by greater communication and collaboration between regulators, and by the sharing of analyses and the rationale behind decision-making^38^.

One solution proposed by meeting participants was to develop an evidence framework that provides a set of objective criteria for ascribing levels of confidence in CoP as predictors of efficacy. Such a framework could draw on models established elsewhere for evidence appraisal, including the GRADE framework for assessing the quality of evidence included in systematic reviews.

### Use of CoP by policymakers

Licensing is a key step on the pathway to vaccine utilisation but not the final one. The decision to use a new vaccine product will be made by ministry of health policymakers, generally having sought a recommendation from independent advisory committees such as national immunisation technical advisory groups (NITAGs). In turn, NITAGs are influenced by the recommendations made by global bodies such as the Strategic Advisory Group of Experts on Immunisation (SAGE) and regional immunisation technical advisory committees (RITAGs).

Policymakers share many of the concerns about safety, quality and efficacy as regulators but also consider local usage issues, including programmatic compatibility and likely effectiveness in local populations, including populations such as pregnant women, and people living with HIV, who may not have been included in clinical trials to generate data for licensure. Data are needed to inform prioritisation between competing priorities, and policy makers use standardised tools to help guide decision making such as the GRADE framework for assessing evidence^39^. However, lack of efficacy data from local settings and populations creates uncertainty about likely vaccine effectiveness, delaying decision making and ultimately use of potentially effective vaccines. Vaccine efficacy can be significantly reduced in some settings, e.g. vaccine efficacy against severe rotavirus diarrhoea in high-income countries was 90.6% compared to just 46.1% in sub-Saharan Africa^40^. Differences in efficacy between low and high mortality settings, with concomitant differences in seroconversion and antibody titres such as those seen with rotavirus vaccines, and lack of mucosal correlates or strong evidence that proposed correlates (serum IgA or neutralising antibodies) are predictive of rotavirus vaccine efficacy mean CoP data are rarely used to inform policy decisions for this vaccine, and policymakers will rely on efficacy data from local populations to inform decisions.

In some cases, efficacy data may be available from similar populations to inform policymaking. If they are not, CoP could provide a tool for assessing likely protection in local populations if the strength and reliability of the association between CoP and efficacy within the population of interest is well established. However, lack of resources can hamper efforts to generate the required data in local populations, which in turn can delay decision making by policy makers whilst additional studies were carried out. For example, SAGE was initially unable to make recommendations on the malaria vaccine RTS,S/AS01 because of insufficient evidence for policy decisions (e.g. whether it was operationally feasible to deliver the vaccine as a 4-dose regimen), even though the data provided were sufficient for regulatory approval, and recommended a pilot implementation programme to be conducted to gather additional data to inform policy decisions^41^. As with regulators, policymakers need to evaluate not only this uncertainty but also the impact of not recommending introduction, so also need to conduct benefit-risk analyses. By incorporating evidence needs of policymakers into early clinical development as the potential to reduce time between vaccine licensure and introduction.

Early engagement of developers with policymakers can help to clarify data needs and alert developers to data gaps, enabling the appropriate studies to be built into clinical development plans, and avoid delays in the formulation of policy recommendations for vaccine use. The value of early engagement and communication between vaccine stakeholders was demonstrated during the COVID-19 pandemic, when SAGE established a COVID-19 vaccine working group with a wide range of global and regional stakeholders, supporting discussions with vaccine developers on evidence needs of regulators and policymakers. With a similar aim, resources such as WHO’s draft Evidence Considerations for Vaccine Policy (ECVP) can provide guidance on integrated evidence needs across regulatory decision-making and policymaking. This has been applied to assess the likely policy-related evidence needs on TB vaccines, in advance of phase III trials, and again emphasizes the importance of co-ordination and communication between stakeholders^42^.

Policy-makers indicated that more emphasis needs to be given to post-licensure studies designed to generate effectiveness and additional safety data as well as consideration of additional data that should be gathered to enable local decision making. Immunogenicity studies could be nested in larger post-licensure studies to provide locally specific data on CoPs. CoP data could be used by policymakers to assess likely differences in vaccine effectiveness between settings. Although phase IV effectiveness studies can provide rich data on vaccine performance to inform policymaking, such studies can be difficult to carry out in some LMICs due to resource shortages, which require prioritisation of health interventions and, within immunisation programmes, of individual vaccines. Ensuring LMIC sites are included in clinical trials to generate efficacy data from disease affected settings could help to reduce these uncertainties. With well-established CoP, local immunogenicity studies could also make an important contribution to national decision-making and could accelerate vaccine development and introduction.

Finally, development of a practical tool that provides guidance on when it is appropriate to use CoPs, based on their intended purpose, and what the evidence requirements are at different stages of decision-making would be valuable to support decision-making.

Correlates of protection have great potential to accelerate vaccine development, licensure and use, are of value to a range of stakeholders and can contribute to multiple aspects of vaccine design, clinical evaluation, licensing and policymaking. However, the use of CoP data is hampered by lack of clarity on which data are needed, how it should be collected and presented, and when and how it is used by different stakeholder groups. Lack of standardisation in assays, protocols and sampling adds to the difficulty of using CoP data. In addition, there are significant gaps in current knowledge of both mucosal and cellular immunity leaving reliance on serum-based markers. There is also a lack of clarity on how to weigh the strength of evidence that a particular immune response truly acts as a predictor of efficacy, with a lack of specific tools to evaluate the relationship between responses and protection.

Discussion in the workshop was used to draft a “data purpose matrix” for correlates of protection data, identifying for vaccine objective, the stakeholder who will use the data, the purpose of such data and which decisions it will be used to inform, and finally the requirements of such data, in order for it to be used by stakeholders (Table 2). Such a framework could be used as a tool to prioritise and coordinate data collection to enable the use of CoP in vaccine development and ensure that data to inform development, licensure and implementation decisions is included in clinical development plans and reduce delays in introducing effective vaccines to target populations. As an exemplar, this was applied to development of vaccines for Group B Streptococcus, to illustrate how the matrix could be used to identify data gaps and prioritise data collection.

All stakeholders were in agreement that there was a critical need for harmonised approaches to data collection including the use of standardised protocols, reagents and assays so that data are reproducible and can be compared and pooled. Several international collaborations have been established to coordinate the development of standardised assays and reagents to define and validate CoP for particular pathogens. These include the GASTON consortium for group B streptococci, which includes academic and industry partners.

Understanding the role played by different immune effectors requires appropriate quantitative assays. For antibodies, binding assays are the simplest to perform but may not necessarily be indicative of an antimicrobial protective function e.g. pneumococcal antibodies may be measured by ELISA, but protection relies on opsonophagocytic activity, and the relationship between the two needs to be defined^43^. Functional assays, which provide a measure of antibody-mediated immune mechanisms, are likely to give a more reliable assessment of protective activity (although correlations are often seen between binding and functional antibody data). T-cell assays are generally harder to perform and to standardise, contributing to a scarcity of T-cell data relative to antibody data in the correlates field, and this was highlighted as a gap by stakeholders for many pathogens.

Assays typically use blood or plasma samples, which are the most convenient to collect. This is not ideal, however, as many initial infections typically occurs at mucosal surfaces (e.g. respiratory or gastrointestinal tract), which have their own unique forms of immune protection. Mucosal immunity is poorly understood but likely to be particularly important as first line of defence against initial infection. Some studies have attempted to map associations between mucosal and systemic responses so that the latter can be used to infer the former. More research is needed into mechanisms of protection at mucosal surfaces and associations between mucosal and systemic immune responses, with guidance established on best practices for mucosal sampling and analysis, as standardising the sampling is challenging.

For each pathogen, developers, regulators and policymakers need to work collectively to identify the most important roles for correlates of protection for different aspects of licensure and approval, as well as the associated evidence needs. Well-designed CoP studies could be used to identify and validate biomarkers for immunobridging, which has the potential to accelerate development and approval of additional vaccines once an efficacious product is licenced. Such studies could transform development of vaccines for high burden diseases such as TB for which new efficacious products are urgently needed. The inclusion of both policymakers and regulators will help to achieve consensus on end-to-end evidence needs. Greater dialogue and collaboration across regulators, including those in LMICs, could help prevent duplication of effort, maximise use of expertise and resources, and ensure greater consistency in practice in relation to CoP. Early engagement between developers and regulators could help to clarify clinical development plans Similarly, early engagement with policymakers could ensure that their evidence needs are factored into clinical development plans.

A framework for evaluation of evidence related to CoPs would be helpful to ensure objective criteria are used consistently to assess the acceptability of CoP-related data in licensing decision-making and to provide guidance on how the evidence in support of CoPs can be strengthened. Using CoPs to secure approval for vaccines against novel targets is a very high hurdle, but CoPs could be critical when it is difficult to obtain efficacy data through field trials. An agreed evidence framework could provide an objective basis for discussing evidence needs in relation to CoP.

All stakeholders have highlighted the need for consistency in data collection and analysis to build relevant and reliable bodies of data on which decisions can be made using CoP data. Collaborations between stakeholders should be encouraged to promote agreement on selection of samples for comparative analysis, with the use of standardised reagents where available to allow comparability of data. Co-ordination between stakeholders on which data are needed to inform decisions based on CoP data could be facilitated by the use of tools such as the draft data purpose matrix presented here. Communication between stakeholders could reduce evidence gaps, time taken to produce required data packages and inconsistencies in decision-making between different groups.

By building collaborations between stakeholders, co-ordination to generate reliable and consistent data sets and communication between those who generate CoP and those who can use it to inform decision-making, the efficiency of vaccine development can be greatly improved. Such synergistic approach has the potential to unlock development of vaccines through improving our knowledge of what constitutes protective immunity in communities most affected by infectious diseases.
